# Targeting brain tumor cAMP: the case for sex-specific therapeutics

**DOI:** 10.3389/fphar.2015.00153

**Published:** 2015-07-28

**Authors:** Nicole M. Warrington, Tao Sun, Joshua B. Rubin

**Affiliations:** ^1^Department of Pediatrics, Washington University School of MedicineSt Louis, MO, USA; ^2^Department of Anatomy and Neurobiology, Washington University School of MedicineSt Louis, MO, USA

**Keywords:** sex differences, cAMP, PDE, primary cilia, brain tumors

## Abstract

A relationship between cyclic adenosine 3′, 5′-monophosphate (cAMP) levels and brain tumor biology has been evident for nearly as long as cAMP and its synthetase, adenylate cyclase (ADCY) have been known. The importance of the pathway in brain tumorigenesis has been demonstrated *in vitro* and in multiple animal models. Recently, we provided human validation for a cooperating oncogenic role for cAMP in brain tumorigenesis when we found that SNPs in *ADCY8* were correlated with glioma (brain tumor) risk in individuals with Neurofibromatosis type 1 (NF1). Together, these studies provide a strong rationale for targeting cAMP in brain tumor therapy. However, the cAMP pathway is well-known to be sexually dimorphic, and SNPs in *ADCY8* affected glioma risk in a sex-specific fashion, elevating the risk for females while protecting males. The cAMP pathway can be targeted at multiple levels in the regulation of its synthesis and degradation. Sex differences in response to drugs that target cAMP regulators indicate that successful targeting of the cAMP pathway for brain tumor patients is likely to require matching specific mechanisms of drug action with patient sex.

## Brain tumors and camp: Some basics

Soon after its discovery, it became clear that cAMP was a ubiquitous second messenger and that normal physiology was dependent upon precise regulation of its synthesis and degradation. As a corollary, investigations rapidly ensued to determine whether pathology was associated with dysregulation of cAMP levels. In 1971, multiple studies were published that associated differences in cAMP levels with differences between normal and cancer cells (Heidrick and Ryan, 1971; Johnson et al., 1971; Otten et al., 1971). Elevated levels of adenylate cyclase (ADCY) activity and cAMP levels were associated with normalization of cell morphology, restoration of contact inhibition and reduced growth rates in neoplastic cells. Subsequently, an inverse relationship between cAMP levels and tumor grade was established for several types of brain tumors (Furman and Shulman, 1977). High ADCY activity and cAMP levels were found in benign brain tumors, while lower ADCY activity and cAMP levels were correlated with greater degrees of malignancy.

Today, there is good reason to think that elevating cAMP will be an important therapeutic for brain tumors, and here, we will review key data validating cAMP as a target in the treatment of brain tumors. We will also discuss the significant sexual dimorphism in this pathway. Evidence for sex-specific responses to drugs that target cAMP indicate that knowledge of sex differences must be incorporated into preclinical and clinical investigations if cAMP is to be successfully targeted in the treatment of brain tumors. It is our perspective that targeting cAMP in the treatment of brain tumors will be a critical test case for the importance of sex-specific treatment of cancer.

## Adenylate cyclase and tumorigenic mechanisms

**Figure 1:**
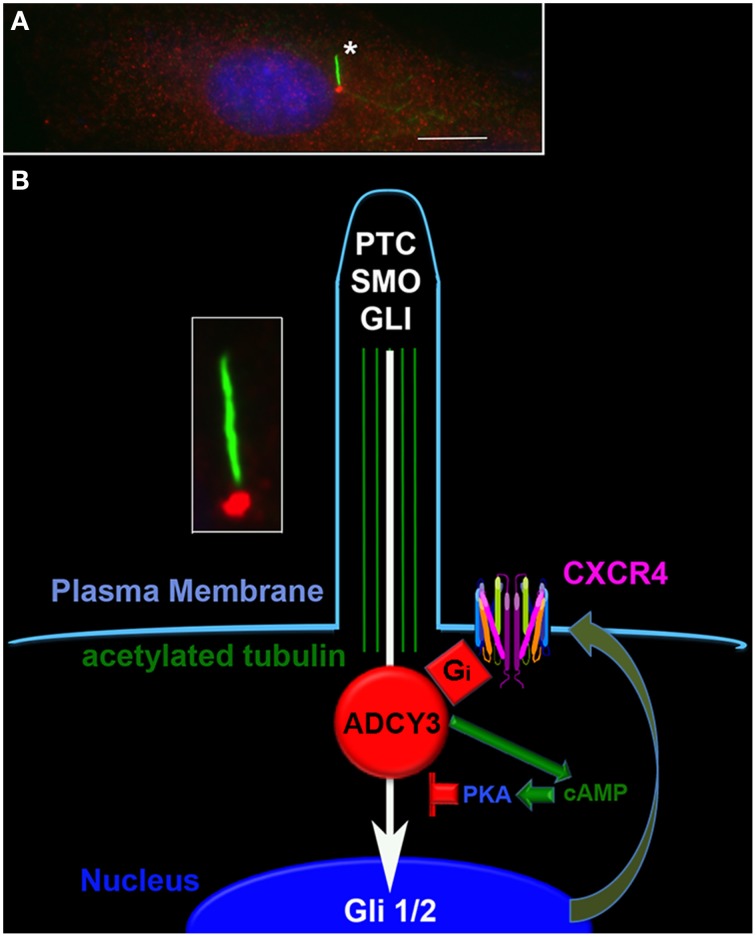
**Cyclic AMP signaling at the primary cilium regulates sonic hedgehog signaling**. **(A)** The primary cilium (asterisk, green) in a medulloblastoma cell is clearly visible under direct immunofluorescence using an antibody directed against acetylated tubulin (Sigma). An antibody directed against ADCY3 (red, Santa Cruz) reveals its localization at the base of the primary cilium. The nucleus is counterstained blue with DAPI. Scale bar equals 10 microns. **(B)** Schematic of the primary cilium indicating the potential interactions between the sonic hedgehog and the CXCR4 pathways. The inset is the cilium from **(A)**. In the schematic are shown the plasma membrane (pale blue) with surface localized CXCR4, acetylated tubulin within the cilium (green), ADCY 3 at the base of the cilium (red), sonic hedgehog signaling components (white), CXCR4 (multi-colored), and the nucleus (dark blue). Activation of CXCR4 results in inhibition of ADCY3, local decreases in cAMP levels, decreased PKA activation and enhanced GLI localization to the nucleus. Increased sonic hedgehog signaling results in increased surface localization of CXCR4.

Differences in cAMP synthesis arise through differences in expression, subcellular localization and activation of nine different membrane bound ADCYs and one soluble ADCY (Cooper and Tabbasum, 2014). Variation in stimulation and inhibition by heterotrimeric G protein subunits, calcium, multiple protein kinase C isoforms and calcium/calmodulin-dependent protein kinase results in unique regulatory “codes” for activation of different ADCY isoforms (Sunahara and Taussig, 2002; Cooper and Tabbasum, 2014). These non-overlapping mechanisms of regulation potentially create alternate landscapes for the interaction between primary oncogenic events in brain tumorigenesis and total ADCY activity. Medulloblastoma and the localization of ADCY3 to the base of the primary cilia (Figure 1A), provides a compelling illustration (McIntyre et al., 2015).

Medulloblastoma is the most common malignant brain tumor of childhood. There are currently four recognized subtypes of medulloblastoma, and one involves mutational activation of the sonic hedgehog (SHH) pathway (Gibson et al., 2010; Northcott et al., 2010; Cho et al., 2011; Kool et al., 2012). Sonic hedgehog signaling involves the activation of a receptor and signaling complex within the primary cilium (Figure 1B). Activation of the pathway results in translocation of the transcription factor Gli2 to the nucleus (Ruat et al., 2012). This key event is blocked by cAMP and protein kinase A (PKA), possibly through stabilization of the complex between Gli2 and Suppressor of Fused (Sufu) (Tuson et al., 2011; Mukhopadhyay et al., 2013). The localization of ADCY3 and cAMP generation to the base of the primary cilium provides a potent and localized mechanism for inhibition of sonic-induced transcription.

A number of experimental findings suggest that the positioning of ADCY3 in primary cilia anatomy may support a cooperative role for cAMP regulation in SHH pathway dependent medulloblastoma-genesis. Sonic hedgehog driven medulloblastoma arises from the cerebellar granule neuron lineage (Oliver et al., 2005). Two key G protein-coupled receptors, PAC1 and CXCR4, are expressed in normal and neoplastic derivatives of this lineage, and function to elevate and suppress cAMP levels, respectively (Klein et al., 2001; Nicot et al., 2002; Rubin et al., 2003). Each pathway is known to powerfully regulate SHH signaling in a cAMP dependent fashion. Mice with combined deficiency of the genes for the SHH receptor, *Patched* (*Ptc*), and the PAC1 ligand, *Pituitary Adenylate Cyclase Activating Peptide (PACAP)*, have increased incidence of medulloblastoma compared to mice with *Ptc* deficiency alone (Lelievre et al., 2008). The effect of PAC1 activation was recently demonstrated to involve inhibition of ciliary translocation of Gli2 in a PKA-dependent manner (Niewiadomski et al., 2013). In related studies, deletion of the alpha subunit of the stimulatory heterotrimeric G protein G_s_ also resulted in SHH-driven medulloblastoma (He et al., 2014). Together these genetically engineered mouse models indicate that diminution in the ability to elevate cAMP levels within the granule neuron lineage is permissive for tumorigenesis in a SHH-dependent fashion.

Consistent with these findings are studies exploring the relationship between the SHH pathway and the Gα_*i*_-coupled receptor, CXCR4. Levels of CXCR4 expression identify two subgroups within the SHH subtype of medulloblastoma (Sengupta et al., 2012). Maximal tumor growth in murine and human models of SHH subtype medulloblastoma is dependent upon co-activation of SHH and the CXCR4 pathway. Surface localization of CXCR4 and consequently, CXCR4-mediated Gα_i_ activation and cAMP suppression, is stimulated by the SHH pathway, creating a positive feedforward loop for suppression of cAMP levels and activation of Gli-mediated transcription (Sengupta et al., 2012). Blockade of CXCR4 signaling, and elevation of cAMP levels, with specific small molecule antagonists has potent anti-tumor effects in intracranial xenograft models of SHH driven medulloblastoma (Rubin et al., 2003; Yang et al., 2007; Barone et al., 2014).

## Phosphodiesterases and brain tumors

While modulation of ADCY activity coordinates cAMP production with activity in other signaling pathways and provides some spatial localization for cAMP production, finer aspects of the compartmentalization of cAMP signaling occur through subcellular localization and regulated activity of phosphodieseterases (PDE) (Bender and Beavo, 2006). Overexpression of the cAMP specific PDEs (PDE4, 7, and 8) is a frequent event in cancer, including brain tumors (Goldhoff et al., 2008; Brooks et al., 2014; Dong et al., 2015). An examination of 37 human pediatric and adult brain tumor specimens including astrocytomas, medulloblastomas, oligodendrogliomas, ependymomas, and meningiomas all exhibited high levels of PDE4A expression, primarily in tumor cells (Goldhoff et al., 2008). Furthermore, overexpression of a super-short, brain specific isoform of PDE4A, PDE4A1, accelerated brain tumor growth in mice bearing intracranial xenografts of human glioma cells (Goldhoff et al., 2008). Inhibition of PDE4 activity with the drug Rolipram inhibited growth in those tumors and in mouse xenografts of human medulloblastoma cells (Yang et al., 2007; Goldhoff et al., 2008). Gene expression profiling of medulloblastoma indicates that cAMP signaling and altered expression of *PDE1C* and *PDE4B* is a characteristic feature of Group D tumors (Northcott et al., 2010).

PDE7B is another cAMP specific phosphodiesterase that is frequently upregulated in glioblastoma (GBM) and is negatively correlated with survival (Brooks et al., 2014). Among the four molecular subtypes of GBM, PDE7B is expressed at the highest levels in Classical, followed by Neural, Mesenchymal, and Proneural subtypes (Brooks et al., 2014). Increased expression of PDE7B was observed in a subset of tumor cells with enhanced tumor initiating capacity, and overexpression of PDE7B in a U87 intracranial xenograft model of GBM transformed the typical circumscribed pattern of intracranial U87 growth into a highly invasive one. These observations suggest that similar to the case of PDE4A1, PDE7B, and cAMP suppression may be critical mediators of tumorigenic mechanisms in GBM, particularly in the Classical and Neural subtypes (Brooks et al., 2014).

Phosphodiesterase expression can be regulated by microRNAs (mirs) and this mechanism has been correlated with tumor biology and therapeutic responses. In diffuse large B cell lymphoma, decreased mir-124 expression led to increased PDE4B expression and subsequent insensitivity to glucocorticoid treatment (Kim et al., 2015). In GBM, mir-33a expression has a negative prognostic effect and is necessary for maintenance and self-renewal of the tumor-initiating cell population. This critical function of mir-33a was dependent upon its direct regulation of PDE8A and UV Radiation Resistance Associated Gene (UVRAG) and their downstream mediators, PKE, and Notch, respectively (Wang et al., 2014). Together with data regarding PDE7B functions in GBM, these findings indicate that the cAMP pathway is essential for tumor initiating cell function. This is a key consideration for promoting cAMP elevating approaches for brain tumor treatment.

The large number of PDE isoforms that are generated from 21 different genes in 11 different families provides for exquisite specialization in cAMP signaling through the formation of diverse PDE signalosome complexes (Azevedo et al., 2014). Phosphodiesterase signalosome complexes are comprised of PDEs in association with scaffolding proteins such as AKAPs, and regulators of cAMP signaling like EPACs at specific subcellular sites that allow for the precise localization of cAMP gradients and subcellular compartmentalization of cAMP signaling. For example, PDE8A complexes with AKAP and Raf-1, a potent activator of MAPK signaling to inhibit PKA mediated inactivation of Raf-1 and MAPK signaling (Brown et al., 2013). PDE4 isoforms are targeted to specific subcellular compartments by unique amino termini. For example, PDE4A1 contains an amino terminal TAPAS-1 domain that localizes it to the trans Golgi complex (Baillie et al., 2002), suggesting that modulation of cAMP levels and its downstream effectors in this domain are critical for PDE4A1's role in brain tumor growth. The peri-Golgi domain is in close proximity to the centrosome and these regions are thought to share a cAMP pool (Verde et al., 2001; Terrin et al., 2012). Modulation of this pool by displacement of localized PDE4D3 has been shown to directly induce cell-cycle arrest (Terrin et al., 2012). Interestingly, protein kinase A is localized to this PDE4D3 signalosome by interacting with AKAP9, which is altered in expression in medulloblastoma (Northcott et al., 2010).

## Cyclic AMP plays a cooperating role in brain tumorigenesis

The work of He et al. (2014) and Lelievre et al. (2008) indicate that AC-mediated cAMP regulation plays an important cooperating role in the genesis of medulloblastoma. The use of genetically engineered mouse models (GEMM) of other brain tumors has also confirmed the importance of PDE activity to brain tumorigenesis. In a GEMM of Neurofibromatosis type 1 (NF1), we found that gliomas could be induced by creating foci of PDE-driven reductions in levels of cAMP (Warrington et al., 2010). NF1 predisposes affected individuals to a number of neoplasms in the central and peripheral nervous systems and other tissues. The most common central nervous system tumor is a low-grade astrocytoma of the optic pathway that most commonly affects children less than 10 years of age (Rubin and Gutmann, 2005). The factors that dictate the temporal and spatial distribution of these tumors are not completely understood. However, there is a clear requirement for the combined effects of: (1) homozygous loss of neurofibromin function in tumor progenitors, (2) heterozygous loss of neurofibromin function in stromal cells of the microenvironment, and (3) other factors specific to the most commonly affected brain regions, such as differences in stem cell populations or growth regulatory/differentiation pathways. Neurofibromin functions as a negative regulator of RAS, and increased RAS activity is accepted as the primary driver of low-grade gliomagenesis through its activation of ERK and AKT pathways (Ratner and Miller, 2015). However, the additional requirements for tumorigenesis indicate that complete loss of *NF1* and hyperactivation of RAS in tumor progenitors is not sufficient for gliomagenesis and that cooperating molecular events must be at play. We described an “oncogenic” mode of CXCR4 signaling that we hypothesized would be a cooperating event in NF1-associated gliomagenesis (Rubin, 2009). We defined this mode of CXCR4 signaling as a loss of receptor desensitization and capacity for sustained suppression of cAMP levels (Warrington et al., 2007; Sengupta et al., 2012; Woerner et al., 2012). The loss of desensitization was the result of ERK dependent phosphorylation and inhibition of GRK2, which we showed resulted in decreased CXCR4 phosphorylation and sustained suppression of cAMP in response to receptor ligation with CXCL12 (Warrington et al., 2007). As we had also shown that CXCL12 was abundant in low-grade gliomas in tumor-associated endothelial cells, microglia, and entrapped neurons, we hypothesized that low levels of cAMP would promote tumor formation. We tested this hypothesis by forcing expression of PDE4A1 in the brains of NF1 GEMM. This model has a highly stereotypical pattern of tumor formation involving the optic pathway alone, and we postulated that if cAMP suppression was a key cooperating event, then expression of PDE4A1 in the cortex would result in the formation of “ectopic” cortical tumors. We chose PDE4A1 for these experiments because the PDE4 family has been shown to be responsible for of the bulk of the cAMP hydrolyzing activity in cells (Conti et al., 2003), and PDE4A1 is a brain specific, super short isoform of PDE4A (Huston et al., 2006). This super short form lacks the regulatory and protein-complexing domains present in longer PDE4 isoforms. Therefore, the effects of PDE4A1 overexpression can be directly attributed to increased cAMP hydrolysis. We found that PDE4A1 expression resulted in foci of decreased cAMP levels that were significantly correlated with the genesis of cortical “ectopic” tumors (Warrington et al., 2010). Furthermore, inhibition of PDE4 with Rolipram blocked the growth of spontaneous tumors in this GEMM (Warrington et al., 2010). Thus, in both medulloblastoma and NF1-associated glioma, primary oncogenic events are mechanistically complemented by decreased levels of cAMP.

Abnormal PDE expression and activity has also been implicated in chronic lymphocytic leukemia (Zhang et al., 2008), lung cancer (Pullamsetti et al., 2013) and colon cancer (McEwan et al., 2007). Similar to the case for polymorphisms in ADCY and cancer risk, single nucleotide variations in PDE8A and PDE11A have been associated with adrenocortical carcinoma (Oliver et al., 2005) as well as cancer of the testes (Klein et al., 2001) and prostate (Nicot et al., 2002).

## Cyclic AMP as a target for brain tumor therapy

The clear correlation between low levels of cAMP and enhanced brain tumorigenesis, brain tumor grade and brain tumor growth, has naturally prompted efforts to develop cAMP elevating approaches to brain tumor treatment. Early efforts utilizing cAMP analogs like 8-chloro-cAMP were associated with dose-limiting toxicities (Propper et al., 1999). Subsequent clinical trials of targeted agents with potential to modulate cAMP levels include G protein coupled receptor (GPCR) agonists and antagonists, as well as stimulators and inhibitors of ACs and PDEs. Notable among the potential clinically available GPCR antagonists that can elevate cAMP are the CXCR4 antagonists AMD3100 (Plerixafor, Genzyme) and POL6326 (Polyphor). AMD3100, AMD3465, and POL5551, also CXCR4 antagonists, have all been shown to block intracranial brain tumor growth in several experimental models, and this activity was correlated with the elevation of cAMP levels in models of GBM and medulloblastoma (Rubin et al., 2003; Yang et al., 2007; Barone et al., 2014). Key to CXCR4 antagonism is the blockade of critical tumor-stromal interactions that occur within the specialized perivascular stem cell niche (PVN) (Rao et al., 2012). In combination with Avastin, POL5551 was shown to block both the formation of new PVN and the function of existing PVN to maintain brain tumor stem cells (Barone et al., 2014).

More specific manipulation of cAMP levels is possible through the use of agents and strategies to increase cAMP synthesis or decrease its degradation. Fundamental principles of pharmacology indicate that inhibition of degradation can have more potent and stable effects on cAMP levels than stimulation of its synthesis (Bender and Beavo, 2006). Consequently, PDE inhibition as a strategy for the therapeutic elevation of cAMP has a long history (Sengupta et al., 2011). Early efforts involved general PDE inhibitors like pentoxyphylline or caffeine, which have both pan-PDE inhibitory and adenosine agonist activities. Non-specific PDE inhibition for cancer applications was complicated by excessive toxicity. As the full breadth of PDE genetics, biochemistry and cell biology became apparent, non-specific approaches gave way to targeted inhibition of specific PDE families. Among the most productive efforts has been the development of a repertoire of PDE4 antagonists. In fact effective PDE4 antagonists for brain tumor therapy may already be clinically available for other applications such as COPD (Sengupta et al., 2011).

## Sex differences in the cAMP pathway demand a sex-specific approach to its targeting

Drug selection for cAMP elevation should proceed carefully and patient selection should be precise. In this regard it will be critically important to recognize the growing body of evidence for significant sex differences in the pathophysiology of human disease and how these might translate into sex differences in therapeutic responses to targeted agents. Sexual dimorphism in the cAMP pathway, and in PDE activity specifically, is likely to impact on the efficacy and appropriateness of specific inhibitors for individual patients.

**Figure 2:**
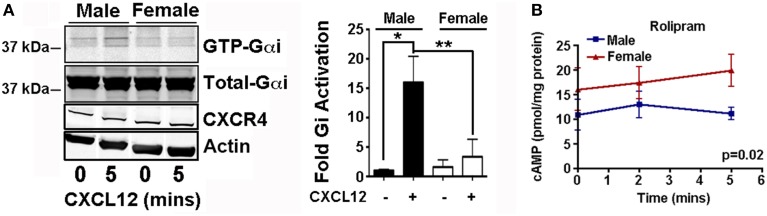
**Sex differences in the cAMP pathway**. **(A)** Activation of Gαi was measured by Western blot for the GTP loaded and total Gαi forms using a kit from Neweast Biosciences as described (Sengupta et al., 2012). Male and female *Nf1*^−∕−^ astrocytes prepared as described (Warrington et al., 2015), serum starved for 48 h, and treated with vehicle (PBS) or CXCL12 (0.1 ug/ml, 5 min). Shown is a representative Western blot and a plot of the fold differences relative to the basal male condition, in the mean and SEM of the fraction of GTP loaded (active) to total Gαi. *N* = 4. ^*^p < 0.05, ^**^p < 0.005 as determined by two-tailed *t*-test. Accompanying Western blots of CXCR4 with actin loading control indicate that differences in Gαi activation are not the result of differences in CXCR4 expression. **(B)** Cyclic AMP levels were measured by ELISA as previously described (Warrington et al., 2015) in male and female *Nf1*^−∕−^ astrocytes treated with Rolipram (20 uM). *N* = 3. *P*-value was determined by Two-Way ANOVA.

There is ample evidence that sex differences exist in cAMP levels. In multiple experimental systems, sex differences have been measured in the expression and activity of cAMP regulators and resultant cAMP levels. For example, neurons in the pontine nucleus of the rat exhibit sexually dimorphic response to corticotropin-releasing factor (CRF). When treated with CRF, female neurons exhibit both greater cAMP elevation and greater PKA activation compared to male neurons. This has been proposed to be the consequence of greater coupling between Gαs and the CRF receptor in female neurons (Valentino et al., 2013). We have made similar observations about the coupling between CXCR4 and Gαi in murine astrocytes. Male and female *Nf1*^−∕−^ astrocytes treated for 5 min with CXCL12 (0.1 μg/ml) exhibit significantly different levels of GTP loading of Gαi despite equal expression of CXCR4 (Figure 2A).

In rat myocytes, greater PDE4B expression leads to lower baseline cAMP levels in female cells. This results in decreased activation of PKA, smaller calcium transients and less forceful contractions than in their male counterparts (Parks et al., 2014). Additionally, PDE3B expression is higher in female endothelial cells rendering them more responsive to treatment with a PDE3 inhibitor than male endothelial cells (Wang et al., 2010).

Sex differences in expression and function of cAMP regulators have also been demonstrated in glioma precursor cells derived from a mouse model of NF1. In this model, astrocytes with targeted deletion of neurofibromin *Nf1*^−∕−^ are poised to be tumorigenic in the appropriate setting, which includes requirements for heterozygous loss of neurofibromin in tumor stromal elements and other, as yet to be fully defined factors, that stereotypically promote tumorigenesis in the optic nerves and optic chiasm. Male *Nf1*^−∕−^ astrocytes expressed more *Gnai3* and less *Gnas* transcript than female cells (Warrington et al., 2015). Female *Nf1*^−∕−^ astrocytes expressed significantly lower levels of *Adcy3* and more *Adcy5* levels, and higher *Pde4a1* levels than male astrocytes. In response to treatment with forskolin, a pan activator of AC, in the presence of IBMX, a pan PDE inhibitor, female cells exhibited greater cAMP synthetic capacity, while in cells treated with forskolin alone, males showed greater capacity to activate phosphodiesterases and block forskolin-induced cAMP elevation. These data indicate that sex differences in expression of cAMP regulators render male and female cells differentially sensitive to the effects of drugs that target the activity of cAMP regulators.

Evidence for sexual dimorphism in the cAMP pathway is also evident in human data. A single nucleotide polymorphism (SNP) array analysis of polymorphisms in the cAMP pathway with DNA from individuals with NF1 with and without optic pathway gliomas revealed that polymorphisms in *ADCY8* (AC8) increase glioma risk in female patients with NF1, but are protective against glioma in male patients (Warrington et al., 2015). Reports of sex specific effects of SNPs in *ADCY7* (rs2302717) in alcohol dependence (Desrivieres et al., 2011) and SNPs in *PDE4B* in schizophrenia (Pickard et al., 2007) suggest there is a broader importance to sex differences in cAMP regulation to human disease.

We would propose that therapeutic cAMP elevation is an ideal setting to test the hypothesis that sexual dimorphism in the cAMP pathway will render males and females differentially sensitive to specific cAMP modulating agents. We previously reported that PDE4 inhibition with Rolipram had significant anti-brain tumor effect in multiple brain tumor models (Yang et al., 2007; Goldhoff et al., 2008; Warrington et al., 2010). We performed a preliminary determination of whether the reported sex differences in PDE4 isoform expression in *Nf1*^−∕−^ astrocytes would render male and female *Nf1*^−∕−^ astrocytes differentially sensitive to Rolipram. Male *Nf1*^−∕−^ astrocytes exhibit lower basal levels of cAMP and are insensitive to Rolipram (Figure 2B). In contrast, female *Nf1*^−∕−^ astrocytes exhibit higher baseline levels of cAMP and these levels further increase with Rolipram treatment. Thus, female brain tumor patients may be more responsive to Rolipram treatment than male brain tumor patients.

The multiplicity of agents that can target different levels of the cAMP regulatory pathways from GPCR through AC and PDE should allow for exhaustive determination of how significant the effects of sex are on cAMP regulation and therapeutic responses to cAMP modulation. Only with this kind of knowledge can we hope to harness the full power of cAMP elevating agents to treat cancer.

### Conflict of interest statement

The authors declare that the research was conducted in the absence of any commercial or financial relationships that could be construed as a potential conflict of interest.
